# Association of loneliness and social network size in adulthood with childhood maltreatment: Analyses of a population-based and a clinical sample

**DOI:** 10.1192/j.eurpsy.2022.2313

**Published:** 2022-09-05

**Authors:** Matthias A. Reinhard, Stephanie V. Rek, Tabea Nenov-Matt, Barbara B. Barton, Julia Dewald-Kaufmann, Katharina Merz, Richard Musil, Andrea Jobst, Eva-Lotta Brakemeier, Katja Bertsch, Frank Padberg

**Affiliations:** 1 Department of Psychiatry and Psychotherapy, LMU University Hospital Munich, Munich, Germany; 2 International Max Planck Research School for Translational Psychiatry (IMPRS-TP), Munich, Germany; 3 Department of Clinical Psychology and Psychotherapy, University of Greifswald, Greifswald, Germany; 4 Department of Psychology, Ludwig-Maximilians-University Munich, Munich, Germany

**Keywords:** Adverse childhood experiences, borderline personality disorder, chronic depression, perceived social isolation, psychopathology

## Abstract

**Background:**

Perceived loneliness and objective social network size are related but distinct factors, which negatively affect mental health and are prevalent in patients who have experienced childhood maltreatment (CM), for example, patients with persistent depressive disorder (PDD) and borderline personality disorder (BPD). This cross-diagnostic study investigated whether loneliness, social network size, or both are associated with self-reported CM.

**Methods:**

Loneliness and social network size were assessed in a population-based sample at two time points (Study 1, *N* = 509), and a clinical group of patients with PDD or BPD (Study 2, *N* = 190) using the UCLA Loneliness Scale and the Social Network Index. Further measures were the Childhood Trauma Questionnaire, and standard depression rating scales. Linear regression analyses were applied to compare associations of loneliness or social network size with CM. Multiple mediation analyses were used to test the relative importance of loneliness and social network size in the relationship between CM and depressive symptoms.

**Results:**

In both studies, loneliness showed a stronger association than social network size with CM. This was particularly marked for emotional neglect and emotional abuse. Loneliness but not social network size mediated the relationship between CM and depressive symptoms.

**Conclusions:**

Loneliness is particularly associated with self-reported CM, and in this respect distinct from the social network size. Our results underline the importance of differentiating both psychosocial constructs and suggest focusing on perceived loneliness and its etiological underpinnings by mechanism-based psychosocial interventions.

## Introduction

Loneliness is a pervasive and adverse experience that is strongly linked to the perception of social isolation [1]. Both persistent loneliness and social isolation may have deleterious consequences for physical and mental health [2–4], and represent risk factors for the development and maintenance of psychiatric conditions, such as depression [1, 5–7]. A deeper understanding of the etiological and maintaining factors of loneliness and social isolation remains an important research endeavor with the ultimate aim to optimize prevention and treatment efforts. This may allow to reduce the burden of loneliness and its negative consequences on mental health.

It is helpful to clearly define the entities of loneliness and social isolation in order to validly study these related, yet distinct phenomena [8]: Loneliness is an aversive feeling resulting from the perceived mismatch between existing relationship and subjective social needs [9]. In contrast, social isolation is an objective criterion that describes a reduced number or absence of social relationships, that is, a small social network size [10]. A small social network is neither a necessary nor sufficient condition to elicit feelings of loneliness as also illustrated by only weak to moderate correlations between the two constructs [10, 11]. An individual may even experience self-sufficiency and immersion into a pleasurable flow when being alone (so-called solitude [12]). Therefore, loneliness may result from a specific perception and subjective evaluation of one’s own social network (i.e., “perceived social isolation” [13]) that does not sufficiently “serve to meet basic emotional needs” [8] (p. 283). In this context, it appears promising to investigate where this perception of unsatisfying social relationships stems from.

Possible influential factors for loneliness and social isolation are adverse prior experiences in life. A history of childhood maltreatment (CM) may hinder individuals to establish close relationships with others [14, 15] and to build social networks [16]. Individuals with a history of CM may develop negative expectations that caregivers and others are not available and untrustworthy [17] possibly resulting in increased social fear and avoidance [18]. In addition, CM is associated with increased rejection sensitivity [19]. This anxious expectation of being rejected may lead to social withdrawal and hostility that actually elicits rejection by others resulting in a self-fulfilling prophecy [20, 21]. Finally, an association between CM and difficulties in emotion regulation may further negatively affect social relationships [22]. Indeed, existing studies on this topic found an association of CM with loneliness later in life in clinical samples with different psychiatric disorders (e.g., borderline personality disorder [BPD], persistent depressive disorder [PDD], late-life depression, drug addiction, psychotic disorder) and non-clinical samples [19, 23–29]. These studies suggest that a history of CM may increase the risk to experience loneliness and/or social isolation later in life. However, a clear distinction between these two constructs is missing in the context of CM research.

CM also represents a major risk factor for chronicity and psychopathology, that is, depressive syndromes [30, 31], yet the mechanisms and pathways underlying this relationship are not fully understood [32]. Interestingly, loneliness and social isolation precede depression when assessed longitudinally [7], and therefore may mediate the path from CM to depressive symptoms as has been found for patients with late-life depression [28].

Thus, our main hypothesis here was that CM is positively associated with loneliness and/or negatively with the social network size. For this purpose, we conducted secondary analyses in two studies with (a) a population-based sample and (b) a clinical sample consisting of patients with BPD or PDD particularly characterized by high rates of CM, loneliness, and a small social network size to test the robustness and generalizability of our findings. On an explorative level, the strengths of the associations of different forms of CM with loneliness versus social network size, and the relative importance of loneliness and social network size as potential mediators in the relationship between CM and depressive psychopathology were analyzed across both samples.

## Methods

### Participants

Both studies followed the Declaration of Helsinki and were approved by the Institutional Review Board (IRB) of the Ludwig Maximilians University, Faculty of Medicine, Munich (Study 1: IRB No. 20–118; Study 2: IRB-Nos. 281–11 and 713–15). Participants gave their written informed consent prior to participation. Studies were preregistered on Open Science Forum (OSF; for IRB No. 20–118: OSF.IO/3EVN9) and at the German Clinical Trials Register (DRKS, for IRB No. 713–15: DRKS00019821).

#### Study 1

Data were derived from *N* = 509 population-based participants who were part of an ongoing longitudinal survey into the mental health consequences of the coronavirus disease-2019 (COVID-19) pandemic [33]. Participants were recruited from the general population via social media advertisements and university mailing lists. The inclusion criteria of the study included a minimum age of 18. The prospective online survey assessed psychopathological symptoms, social network characteristics, and loneliness, in addition to other questionnaires at two time points [33]. Of the 509 individuals at baseline, 345 participated in a 10 week follow-up assessment. No significant differences were observed between participants that dropped out and participants that provided data at two time points in terms of sociodemographic characteristics and reported measures (see Table 1). The secure online “LimeSurvey” software with a forced response format and questionnaire block randomization was used in this study. As a compensation for their participation, participants were given the opportunity to win gift vouchers.

**Table 1.** Baseline characteristics of the population-based sample (Study 1) stratified by follow-up participation.

	Non-completers	Completers	*p* value
*n*	164	345	
Age, mean (SD)	29.0 (10.8)	30.6 (11.2)	0.130
Female, *n* (%)	170 (80.6)	303 (77.5)	
Marital status, *n* (%)			
Married	27 (16.5)	61 (17.7)	0.806
Partnership	56 (34.1)	126 (36.5)	
Single	77 (47.0)	149 (43.2)	
Divorced	2 (1.2)	7 (2.0)	
Widowed	2 (1.2)	2 (0.6)	
Employment status, *n* (%)			
Full-time employed	34 (20.7)	79 (22.9)	0.174
Part-time employed	20 (12.2)	52 (15.1)	
Self-employed	4 (2.4)	12 (3.5)	
Student	78 (47.6)	169 (49.0)	
Retired	3 (1.8)	5 (1.4)	
Caregiver	2 (1.2)	0 (0.0)	
Not employed	5 (3.0)	9 (2.6)	
Other	18 (11.0)	19 (5.5)	
Self-reported lifetime diagnoses, *n* (%)			
Depressive disorders	35 (21.3)	83 (24.1)	0.571
Bipolar disorders	2 (1.2)	2 (0.6)	0.821
Psychotic disorders	3 (1.8)	0 (0.0)	0.057
Anxiety disorders	24 (14.6)	38 (11.0)	0.307
Post-traumatic stress disorder	8 (4.9)	18 (5.2)	1
Eating disorders	13 (7.9)	15 (4.3)	0.148
Obsessive–compulsive disorders	0 (0.0)	6 (1.7)	0.208
Addictive disorders	6 (3.7)	3 (0.9)	0.061
Attention-deficit/hyperactivity disorder	7 (4.3)	8 (2.3)	0.35
Somatoform disorders	2 (1.2)	2 (0.6)	0.821
Autism spectrum disorder	1 (0.6)	3 (0.9)	1
Personality disorders	7 (4.3)	7 (2.0)	0.249
Dementia	0 (0)	0 (0)	1
Childhood trauma total	37.7 (14.0)	37.3 (12.4)	0.720
Emotional abuse	9.0 (4.7)	9.0 (4.6)	0.891
Emotional neglect	10.0 (5.0)	9.9 (4.6)	0.921
Sexual abuse	5.8 (2.6)	5.7 (2.6)	0.878
Physical abuse	6.0 (2.3)	5.8 (2.0)	0.308
Physical neglect	6.9 (2.8)	6.8 (2.5)	0.695
Loneliness total	2.2 (0.8)	2.2 (0.7)	0.573
Depression total	12.3 (10.2)	12.0 (10.8)	0.740
Social network size	16.3 (8.6)	15.2 (8.5)	0.163

#### Study 2

A clinical sample was derived from 190 psychiatric inpatients (94 patients with BPD and 96 patients with PDD) who participated in two trials at the Department of Psychiatry and Psychotherapy of the LMU University Hospital, Munich, Germany. Patients were mainly recruited from two wards that specialized in the treatment of patients with BPD and PDD, respectively [34, 35]. Diagnoses were assessed with the German version of the Structured Clinical Interview for DSM-IV (SCID-I/-II) or The Diagnostic and Statistical Manual of Mental Disorders, Fifth Edition (DSM-5) (SCID-5-CV/-PD) by trained and supervised psychologists or psychiatrists. As the German version of the DSM-5 was already available before the release of SCID-5-CV, PDD diagnosis was additionally confirmed with DSM-5 criteria.

### Measures

#### Loneliness

The UCLA Loneliness Scale [36] (German version: [37]) consists of 20 items that measure the frequency and intensity of loneliness. Items are rated on a 5-point Likert scale ranging from 1 (not at all) to 5 (totally). After recoding reversed items, items are averaged to form a mean score. The internal consistency for the German version is high (Cronbach α = 0.89 [37]).

#### Social network size

The Social Network Index (SNI [38]) assesses how many people the respondent meets or talks to at least once every 2 weeks within 12 different domains of social relationships (e.g., parents, friends). The 12 items range from 0 to “7 or more” persons. The number of persons per domain is summarized as a total network size score. This score was used as a dimensional indicator for the extent of social isolation with a smaller network size indicating more social isolation.

#### Childhood maltreatment

The Childhood Trauma Questionnaire (CTQ [39], German version: [40]), consists of 25 items that measure the subjective experiences of emotional, physical and sexual abuse as well as emotional and physical neglect before the age of 18 years. Items are rated on a 5-point Likert scale ranging from 1 (never true) to 5 (very often true). Subscales range from 5 to 25. For the CTQ total score subscales were summed up. Internal consistency of all subscales is high apart from physical neglect (Cronbach α > 0.80 [41]).

#### Depressive symptoms

The population-based sample filled out the Depression, Anxiety and Stress Scale-21 (DASS-21 [42], German version [43]) 10 weeks after measuring loneliness and social network size. In the following, the depression subscale was used, which shows a high internal consistency (Cronbach α > 0.90 [43]). For patients, we used the Beck Depression Inventory (BDI-II [44], German version: [45]) as DASS-21 scores were not available in this sample. BDI-II was measured cross-sectionally and shows a high internal consistency (Cronbach α > 0.84 [46]). The correlation between the depression subscale of the DASS-21 and BDI-II was found to be strong in a German sample (*r* = 0.68 [43]).

### Statistical analyses

Statistical analyses were conducted with R v4.0.3 [47]. There were no missing data at baseline in Study 1. In Study 2, two patients had missing data and were excluded from the specific analyses concerning the social network size. Analyses were performed independently yet identically for the two studies due to different sample sizes and characteristics, sampling methods, and questionnaires. Descriptive statistics are presented in mean and standard deviation. To test our main hypothesis, regression analyses were performed with total CTQ as an independent variable and either loneliness or social network size as a dependent variable. Variables were standardized to ease the interpretation of results. Results were controlled for age and sex. Fisher’s *Z* was computed to compare the strength of correlation coefficients for loneliness versus social network size. In addition, regression analyses were separately repeated for each CTQ subscale as an independent variable. *p*-values were adjusted according to [48]. Finally, multiple mediation analyses were performed to test whether loneliness and smaller social network size mediated the relationship between total CTQ and depressive symptoms. Mediating variables were allowed to correlate in these models.

## Results

### Study 1

Data of 509 participants from the general population (78.2% female, mean age: 30.1 ± 11.1 years) were analyzed. Participants reported a mean score of 2.2 ± 0.8 on the UCLA loneliness scale and an average social network size of 14.8 ± 8.8 people. Loneliness correlated negatively with the social network size (*r* = −0.41, *p* < 0.001). Participants reported an average total CTQ of 37.4 ± 12.9. According to [39], the average reported emotional abuse was low to moderate (mean: 9.0 ± 4.6) as was emotional neglect (mean: 9.9 ± 4.7). The sample reported none to minimal physical neglect (mean: 6.9 ± 2.6), physical abuse (mean: 5.9 ± 2.1), and sexual abuse (mean: 5.7 ± 2.6). The mean DASS-21 depression score was 12.1 ± 10.6, indicating a significant prevalence of depressive symptoms [43].

**Figure 1.**
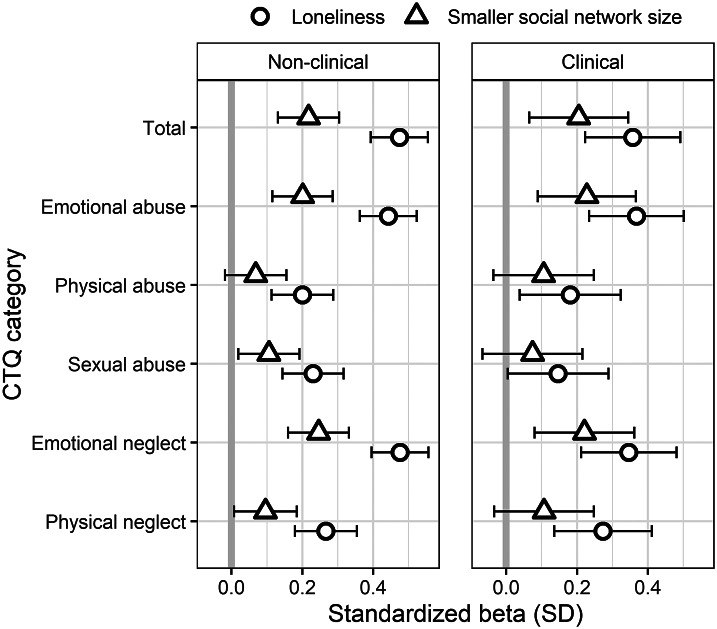
Association of loneliness and smaller social network size with childhood trauma questionnaire (CTQ) in a population-based (non-clinical) and a clinical sample.

Higher loneliness and a smaller social network size were found to be significantly associated with the total CTQ score (see Figure 1 and Table 2). The correlation between loneliness with total CTQ was stronger than of social network size with total CTQ when comparing the strengths of the regression coefficients (Z = 5.7, *p* < 0.001). Furthermore, loneliness and social network size were significantly associated with all CTQ subscales (despite physical abuse). The strongest associations were found for the subscales of emotional neglect and emotional abuse with loneliness.

**Table 2.** Regression analyses for loneliness and social network size as dependent variables and childhood trauma questionnaire (CTQ) total score and subscales as predictors in the population-based (Study 1) and clinical sample (Study 2).

Study 1	Outcome loneliness (*N* = 509)				Outcome social network size (*N* = 509)			
Predictor	Estimate (SE)	*T* value	*p* value	*R* ^2^	Estimate (SE)	*T* value	*p* value	*R* ^2^
Total score	0.47 (0.04)	11.56	<0.001***	0.21	−0.22 (0.04)	−4.92	<0.001***	0.08
Emotional abuse	0.44 (0.04)	10.86	<0.001***	0.19	−0.20 (0.04)	−4.61	<0.001***	0.08
Physical abuse	0.20 (0.04)	4.52	<0.001***	0.04	−0.07 (0.04)	−1.54	0.123	0.04
Sexual abuse	0.23 (0.04)	5.25	<0.001***	0.05	−0.11 (0.04)	−2.40	0.017*	0.05
Emotional neglect	0.48 (0.04)	11.63	<0.001***	0.21	−0.25 (0.04)	−5.61	<0.001***	0.10
Physical neglect	0.27 (0.04)	5.97	<0.001***	0.07	−0.10 (0.05)	−2.12	0.034*	0.05

Study 2	Outcome loneliness (*N* = 190)				Outcome social network size (*N* = 188)			
Total score	0.36 (0.07)	5.25	<0.001***	0.15	−0.20 (0.07)	−2.86	0.005**	0.09
Emotional abuse	0.37 (0.07)	5.42	<0.001***	0.15	−0.23 (0.07)	−3.22	0.002**	0.10
Physical abuse	0.18 (0.07)	2.5	0.013*	0.05	−0.11 (0.07)	−1.46	0.146	0.06
Sexual abuse	0.15 (0.07)	2.03	0.044	0.04	−0.07 (0.07)	−1.03	0.304	0.06
Emotional neglect	0.35 (0.07)	5.06	<0.001***	0.14	−0.22 (0.07)	−3.07	0.002**	0.10
Physical neglect	0.27 (0.07)	3.91	<0.001***	0.10	−0.11 (0.07)	−1.49	0.139	0.06

*Note*: Outcome and predictor variables were standardized to ease interpretation of results. Age and sex (standardized) were included as covariates.

* *p* < 0.05;

** *p* < 0.01;

*** *p* < 0.001 significance level after Benjamini–Hochberg *p*-value correction.

**Figure 2.**
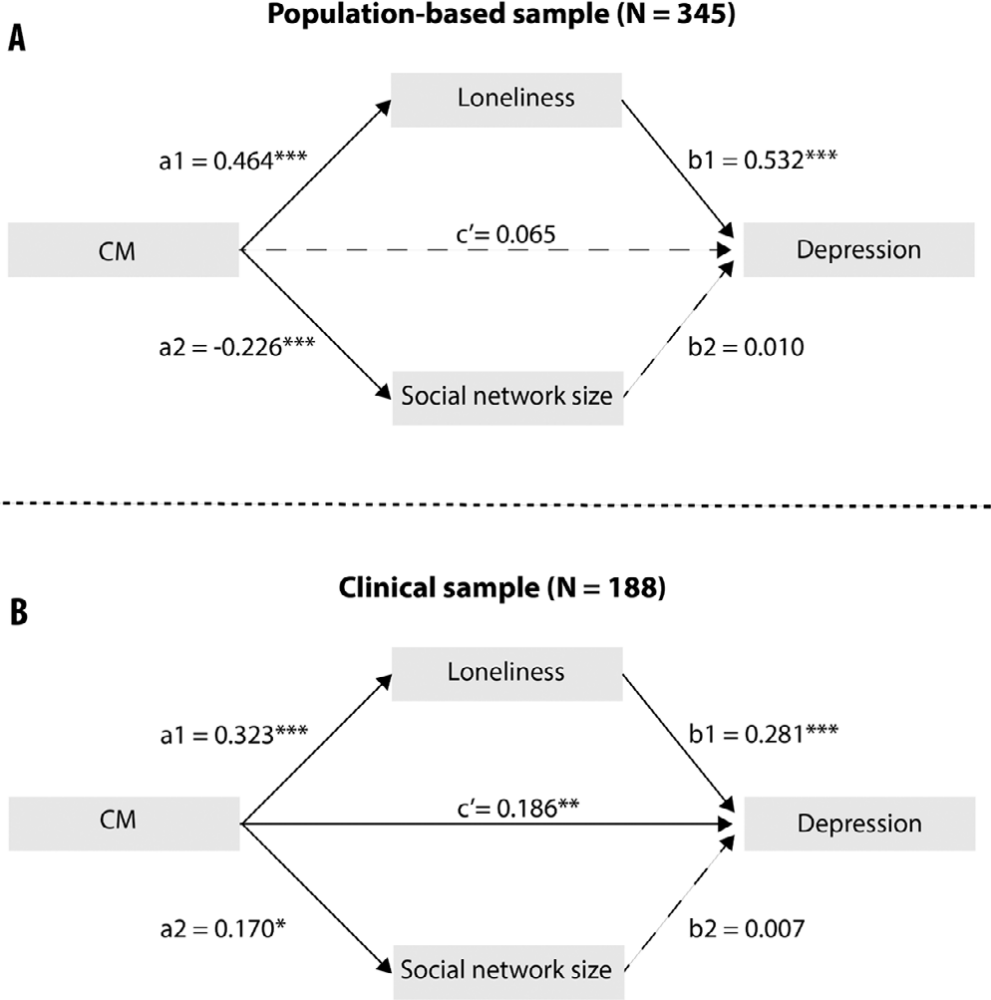
Mediation model of childhood maltreatment (CM) as predictor of depression mediated by loneliness and social network size in Study 1 (A) and Study 2 (B).

Finally, multiple mediation analyses were performed including depressive symptoms in the population-based sample (see Figure 2 and Table 3). Here, loneliness—but not social network size—fully mediated the relationship between CTQ and depressive symptoms at 10-week follow-up.

**Table 3.** Results of multiple mediation analyses with childhood maltreatment as predictor of depression and loneliness and social network size as mediators.

Study 1 (*n* = 345)	Std. point estimate	SE	*p*	CI lower	CI upper	*R* ^2^
DV (after 10 weeks)						
Total (c)	0.309	0.054	<0.001	0.147	0.358	0.31
Total indirect	0.244	0.029	<0.001	0.146	0.260	
Loneliness	0.247	0.030	<0.001	0.147	0.264	
Social network size	−0.002	0.008	0.816	−0.02	0.014	
Direct (c’)	0.065	0.052	0.309	−0.05	0.156	

Study 2 (*n* = 188)	Std. point estimate	SE	*p*	CI lower	CI upper	*R* ^2^
DV						
Total (c)	0.276	0.040	<0.001	0.094	0.251	0.23
Total indirect	0.089	0.019	0.004	0.022	0.097	
Loneliness	0.091	0.019	0.003	0.024	0.097	
Social network size	−0.001	0.009	0.933	−0.02	0.016	
Direct (c’)	0.186	0.043	0.007	0.032	0.201	

*Note:* Depicted are total, total indirect, and direct effects of the different multiple mediation models.

Abbreviations: CI, confidence interval (bootstrapped); DV, dependent variable; Std., standardized.

### Study 2

The clinical sample consisted of 190 psychiatric patients (65.3% female, mean age: 33.3 ± 12.3 years, *n* = 96 patients with BPD, *n* = 94 patients with PDD). Patients reported a mean loneliness level of 2.9 ± 0.8 and a social network size of 9.4 ± 6.4 people. Patients’ loneliness correlated significantly negatively with social network size (*r* = −0.30, *p* < 0.001). Total CTQ was 55.2 ± 17.5. CTQ subscales of the clinical sample ranged from moderate to severe emotional abuse (mean: 14.8 ± 5.7) and emotional neglect (mean: 15.9 ± 5.1) to moderate sexual abuse (mean: 7.6 ± 4.6) and moderate physical neglect (mean: 9.2 ± 3.6) according to [39]. Patients reported minimal to low physical abuse (mean: 7.7 ± 4.0). Mean BDI-II scores were 31.0 ± 10.8 indicating severe depressive symptoms [49].

Both loneliness and social network size were significantly associated with the total CTQ score (see Figure 1 and Table 2). Again, the association between loneliness and total CTQ was stronger than the correlation between social network size and total CTQ (Z = 2.0, *p* < 0.05). Loneliness was significantly associated with all CTQ subscales (except sexual abuse), whereas social network size was only associated with CTQ emotional abuse and neglect subscales.

Mediation analysis revealed comparable results, which were in line with Study 1: Loneliness—but again not social network size—partially mediated the relationship of CM with depressive symptoms (see Figure 2 and Table 3).

## Discussion

The main finding of our studies was the strong association between loneliness and a smaller network size with self-reported CM across two independent samples, that is, a population-based and a clinical sample. Loneliness was particularly associated with emotional neglect and emotional abuse, and regression coefficients between loneliness and CM were significantly larger than coefficients between social network size and CM in both samples. This underlines the importance of clearly defining both constructs and distinguishing the more subjective feeling of loneliness from objective social isolation which can be quantified using the social network size. Regarding their interaction with depressive psychopathology, loneliness but not social network size mediated the relationship between CM and depression in both studies.

Our results of a close relationship between loneliness and CM are in line with a recent meta-analysis that found that individuals with a history of CM feel lonelier than individuals without a history of CM [50]. Therefore, CM may constitute a specific vulnerability factor to experience loneliness later in life via different pathways. For instance, Rokach [51] suggests that growing up in an inadequate or dysfunctional home characterized by emotionally distant or rejecting parents, abuse, and an atmosphere that is generally characterized by upset and unhappiness may lead to developmental deficits as an antecedent of adult loneliness. Individuals with a history of CM may develop difficulties in emotion regulation [22], unhelpful cognitions and schemas [30], and increased rejection sensitivity (i.e., the readily perception, overreaction, and expectation of rejection [21]) that may hinder forming social relationships that offer sufficient social support [14–16, 52]. The experience of CM may even bias the perception of adequate and sufficient social support towards unsatisfying relationships that do not fulfill one’s social and emotional needs.

Regarding specific forms of CM, a history of emotional neglect and emotional abuse seems to be particularly associated with perceived loneliness according to our results which is in line with the meta-analytic findings of [50]. Emotional neglect is the failure of caretakers or parents to satisfy a child’s emotional needs such as belonging. In contrast, emotional abuse refers to humiliating and demeaning behavior by caretakers. Both emotional neglect and abuse could theoretically induce aversive feelings and support assumptions about being abandoned or even rejected and lead to the expectation that others are emotionally not available [17]. The experience of emotional maltreatment and the frequent frustration of emotional needs during childhood may be internalized as deleterious object relations [53] that reduce the quality of later relationships and the ability to feel close to others.

Our findings also underline the importance to investigate differences between apparently related phenomena as loneliness and social network size. In general, associations for loneliness with CM were more pronounced than for social network size and CM. Both loneliness and social network size clearly differ in the extent of subjective content with loneliness representing a rather emotional experience that is linked to psychopathology [19], and social network size a more objective measure, though both are usually assessed with self-report scales. Thus, asking for feelings of loneliness may particularly assess the emotional burden of social isolation and reduced quality of interpersonal relationships, and better reflect the level of interpersonal stress [19]. One could speculate that previous CM as retrospectively assessed by the CTQ has led to negative assumptions about interactions and expectations including potential rejection during development making the individual prone to experience burdensome loneliness, whereas the phenomenon of a reduced social network size is not as directly related to CM.

In addition, only loneliness but not social network size mediated the relationship between CM and depressive symptoms. This result is in line with the findings of Wielaard et al. [28] who found that both, loneliness, and social network size, mediated between CM and late-life depression in a sample of older adults when analyzed in separate mediation models. However, when including both as mediators only loneliness stayed significant [28]. Also in this respect, loneliness may be closer to the spectrum of interpersonal stressors and may be one core factor for the development of depressive symptoms. Indeed, Fried et al. [54] showed in a network model that partner loss mainly affected loneliness, which in turn activated other depressive symptoms. Associations between a variety of psychopathological symptoms, especially depression, and CM are well known [30, 55], yet the exact mechanisms are unclear in how CM unfolds its negative consequences [32]. An insecure attachment style may represent one possible pathway as it has been found that the effect of emotional abuse on depression is partially mediated by an anxious attachment style and in the case of emotional neglect on depression by an avoidant attachment style [56].

The results of our two studies further demonstrate similar association patterns in independent samples that differ in terms of their average CM load, levels of loneliness and social network size, underlining the robustness and generalizability of our findings. Interestingly, the associations of loneliness and social network size with CM were stronger in the population-based compared to the clinical sample. Additional clinical and/or neurobiological factors that contribute to or weaken this association need to be identified. Interestingly, a recent study compared different environmental (CM, social support) and neurobiological factors such as neuroimaging findings and polygenic risk scores (PRS) in depressed patients compared to healthy individuals [57]. Whereas classification accuracy for CM and social support was 71%, it was lower for neuroimaging modalities (ranging between 54 and 55%) and for PRS (58%) underlining the importance of these constructs.

Future research should investigate the mechanistic pathways from CM to loneliness and psychopathology in order to develop tailored treatment options. The interplay of loneliness, a small social network, and psychopathology needs to be further disentangled by taking factors like attachment style, rejection sensitivity, relationship quality, and social support into account. Measuring the effect of specific interventions that unfold over time, for example, addressing unhelpful schemas and expectations, or preventive interventions in children that feel lonely may provide a deeper insight into maintaining factors of loneliness.

Strengths of our study are the replication across independent samples and the parallel assessment of loneliness and social network size addressing the question, which construct shows stronger associations with CM. On the other hand, there are clear limitations: First, a selection bias may have occurred in both, that is, the population-based sample that was recruited via social media during the ongoing COVID-19 pandemic that may have impacted the social network measures, and the clinical sample that consisted of psychiatric inpatients. A cross-diagnostic approach with a larger sample ranging from clinical, to subclinical and nonclinical participants from the general population, may further clarify the association of CM and loneliness. Second, the relatively small clinical sample and partially reduced variance of CTQ subscales hamper further in-depth analysis of our main findings. Third, both studies used self-assessment scales. This may be particularly critical for assessing CM with the CTQ, though this questionnaire is a commonly used instrument for CM. One needs to be aware that the CTQ ask for memories and interpretations related to CM, but does not assess actual records of CM. There is an ongoing debate on the congruence between recorded CM and its retrospective assessment that is driven by the results of recent longitudinal studies. For instance, Baldwin et al. [58] and Newbury et al. [55] reported a poor agreement between prospective and retrospective measures of CM. Similarly, Danese and Widom [30] found that the risk of later psychopathology hardly was associated with objective measures but rather linked to subjective reports even if reports were inconsistent with objective measures. However, the authors also demonstrated that this finding is not caused by a negative autobiographical memory bias due to current psychopathology. Instead, they suggest that it is rather necessary to identify unhelpful cognitions and memories about the self and the environment that may endorse even in the absence of objective maltreatment. These cognitions and memories may be rooted in early adversity and traumatization without representing sound autobiographical information. Nevertheless, they could be an important key to further understanding the role of CM in different perceptual and interpretational processes. Thus, longitudinal studies using documented information and records on CM would be an important step to support our hypothesis.

To conclude, our results suggest a strong association between perceived loneliness and self-reported CM, which appears to be generalizable across populations. CM may increase the vulnerability to experience loneliness later in life which constitutes an aversive inner state closely interacting with the individual psychopathology. Further research investigating causal interactions over the life span, for example, longitudinal studies, is necessary to gain a deeper mechanistic understanding of loneliness, social isolation, and related factors. Focusing on reducing loneliness in psychotherapy may be an interesting approach to buffer the negative consequences of CM on mental health.

## Data Availability

The raw data supporting the conclusions of this article will be made available by the authors, without undue reservation.
